# Auto-inflammatory diseases in ileal pouch patients with NOD2/CARD15 mutations

**DOI:** 10.1093/gastro/gou069

**Published:** 2014-10-12

**Authors:** Darren N. Seril, Qingping Yao, Bo Shen

**Affiliations:** ^1^Division of Gastroenterology/Hepatology, Albany Medical College, Albany, New York, USA; ^2^Departments of Rheumatic and Immunologic Disease, The Cleveland Clinic Foundation, Cleveland, Ohio, USA; ^3^Department of Gastroenterology/Hepatology, The Cleveland Clinic Foundation, Cleveland, Ohio, USA

**Keywords:** auto-inflammation, ileal pouch, pouchitis, restorative proctocolectomy, ulcerative colitis

## Abstract

Pouchitis is common in ulcerative colitis patients undergoing total proctocolectomy with ileal pouch-anal anastomosis, and chronic antibiotic-refractory pouchitis occurs in a subgroup of the patients. Auto-inflammatory diseases are characterized by systemic inflammation, manifesting as periodic fever, rash, arthritis, and serositis. We describe two cases with ulcerative colitis and an ileal pouch, who presented with extra-intestinal manifestations and genetic features atypical for inflammatory bowel disease alone. Case 1 had a spectrum of clinical manifestations including refractory pouchitis, intermittent fevers, polyarthralgia, and pericarditis. Case 2 presented with oral ulcers, migratory oligoarthritis, and periodic papular rash. Genetic testing in both cases revealed mutations of the *NOD2/CARD15* gene, including the IVS8^+158^ mutation commonly detected among patients with *NOD2*-associated auto-inflammatory disease. Both of the patients demonstrated clinical improvement of these diverse systemic complaints following treatment with immunosuppressive and anti-inflammatory therapies.

## Background

Total proctocolectomy with ileal pouch-anal anastomosis (IPAA) is a commonly performed surgical procedure for medically refractory ulcerative colitis (UC) or UC-associated neoplasia [1]. Pouchitis is common after IPAA, with alterations in the pouch microbiota being the major etiological factor. In a subgroup of patients, pouchitis is primarily immune-mediated and is associated with features of autoimmunity [1].

Autoinflammatory diseases (AInD), as typified by familial Mediterranean fever (FMF), have features of systemic inflammation that commonly manifest as fever, rash, arthritis, or serositis of multiple organ systems. AInD are monogenic or polygenic entities, and the disease process is mediated by abnormal innate immunity [2–4]. In addition, some features of AInD correspond to extra-intestinal manifestations (EIMs) of inflammatory bowel disease (IBD) and pouchitis. Indeed, genetic overlap between IBD and AInD has been reported [5–7]. However, the role of auto-inflammation in the pathogenesis of pouchitis—and its contribution to chronic antibiotic-refractory pouchitis (CARP)—are unknown.

## Case 1

We describe a 52-year-old Caucasian woman with UC diagnosed at the age of 37, along with a history of thyroid cancer, treated with thyroidectomy, and supraventricular tachycardia, treated with radio-frequency ablation. Three years after being diagnosed with UC, she developed toxic megacolon and underwent proctocolectomy and a two-stage J-pouch surgery. The post-operative period following the first stage was complicated by anastomotic leakage and pelvic abscess that was surgically drained. Shortly after closure of the ileostomy, she had symptoms of frequency, urgency, blood in stool, and lower abdominal pain. Multiple pouch endoscopies identified ulcers along folds of the pouch body, consistent with pouchitis, as well as erythema, edema, and friability of the anal transition zone consistent with cuffitis. Her diarrhea responded partially to oral metronidazole treatment. She later had persistent pouchitis-attributed symptoms despite oral ciprofloxacin 500 mg b.i.d. and a combination of ciprofloxacin 500 mg b.i.d. and rifaxamin 200 mg q.d. In addition, following ileostomy closure she developed recurrent abscesses in the perianal region on the fourchette of the vagina, and was diagnosed with ano-vaginal fistula treated with placement of a seton. Based on the occurrence of ano-vaginal fistula, the diagnosis of Crohn’s disease (CD)-like condition of the pouch was considered as an alternative to surgical anastomosis-related fistula.

**Figure 1.**
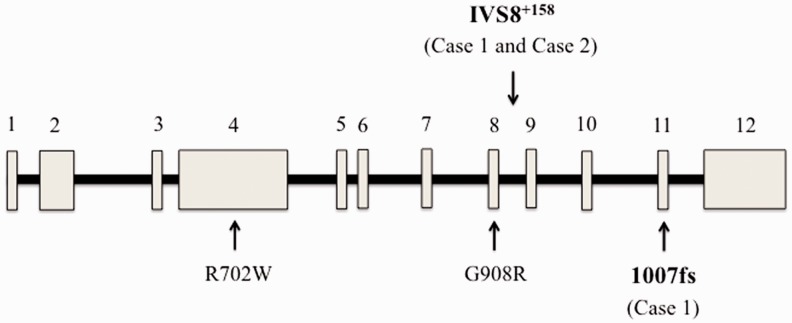
Structure of the *NOD2/CARD15* gene and location of mutations detected in the cases. R702W, G908R, and 1007fs (3020insC) are among the most common variants detected in Crohn’s disease patients, and have also been described in pouchitis. The IVS8^+158^ variant is rare in Crohn’s disease and Blau syndrome, but is characteristic of NOD2-associated auto-inflammatory disorder (NAID). Numbered blocks represent exons, and intervening lines represent introns.

Four years after the pouch surgery, the patient presented with fever, left-sided chest heaviness, and abdominal pain. An echocardiogram showed a moderate pericardial effusion without tamponade, for which she was treated with indomethacin and colchicine. Follow-up echocardiogram showed a smaller but persistent pericardial effusion. Following treatment for pericarditis using indomethacin and colchicine, the patient continued to have intermittent episodes of fever and arthralgia involving the shoulder, low back, and lower extremities. She underwent diverting loop ileostomy for chronic perianal sepsis at the age of 49. She subsequently developed ulceration in the peri-stoma region as well as on her foot. The skin lesions were diagnosed as pyoderma gangrenosum and she was treated with prednisone. Serological testing was positive for anti-nuclear antibody (5.2 optical density ratio), and an elevated C-reactive protein (1.2 mg/dL). Genetic testing for nucleotide-binding oligomerization domain-containing protein 2/caspase recruitment domain-containing protein 15 (*NOD2/CARD15*) revealed two mutations: 3020insC and IVS8^+158^ (Figure 1). A diagnosis of AInD was entertained, based on the results of genetic tests, fevers, serositis, and polyarthralgia. The patient was started on 6-mercaptopurine and celecoxib for the treatment of an AInD, with resolution of the symptoms of fever and arthralgia. She continues to have a diverted ileal pouch, and a permanent end ileostomy has been recommended.

## Case 2

**Figure 2.**
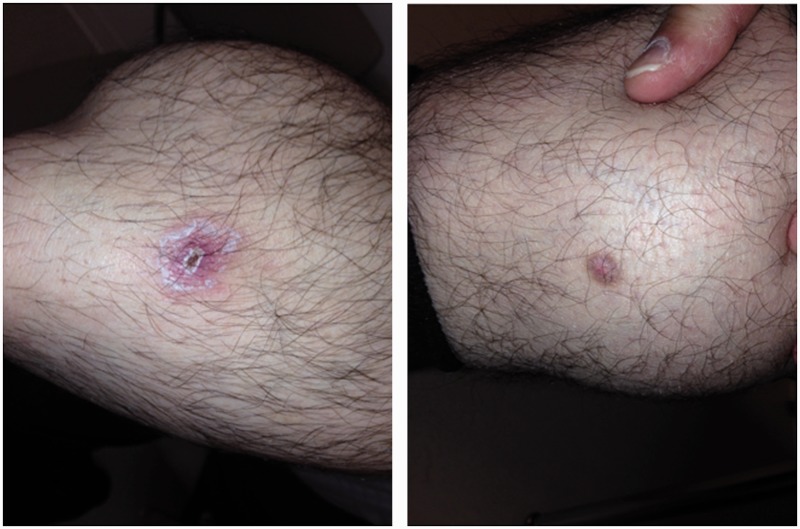
Erythematous papules noted on the lower extremities of the ileal pouch patient in Case 2. The patient also had lesions of the upper extremities and trunk.

A 48-year-old man of Middle-Eastern descent was diagnosed with ulcerative pancolitis at the age of 23. At the age of 39 he underwent two-stage proctocolectomy with J-pouch for corticosteroid-refractory UC and rectal adenocarcinoma. Prior to reversal of an ileostomy, he received adjuvant chemotherapy and the malignancy has remained in remission. Eight years after ileostomy reversal, he presented with fecal urgency and frequency characterized by up to 15 bowel movements per day, with some benefit derived from the use of fiber supplement and diphenoxylate/atropine. Pouch endoscopies, performed yearly following reversal of the ileostomy, were normal. For four years prior to the presentation he noted nearly constant, tender oral cavity ulcers. In addition, he developed intermittent, erythematous, papular rash involving the face, trunk, and extremities (Figure 2). A punch biopsy of the rash revealed acanthosis and hypergranulosis of the epidermis, and a perivascular and interstitial lymphocytic infiltrate of the dermis. The patient also noted severe pains in the joints of the lower extremities, associated with a migratory pattern, joint swelling, and morning stiffness. Laboratory evaluation was notable for normal thyroid stimulating hormone and immunoglobulin G and E levels. In addition, serology was negative for anti-transglutaminase antibody, anti-nuclear antibody, microsomal antibody, rheumatoid factor, and human leukocyte antigen B27. Based on the spectrum of symptoms—including diarrhea, migratory oligoarthritis, and erythematous rash—genetic testing of *NOD2/CARD15* was obtained, revealing the IVS8^+158^ variant (Figure 1). The patient was treated with sulfasalazine and colchicine for the management of AInD.

## Discussion

This is the first report of pouch patients with clinical and genetic features of AInD. The 3020insC mutation is among the most common *NOD2/CARD15* mutations reported in CD, and has also been described in IPAA patients with severe pouchitis [8]. However, 3020insC has not been reported in AInD attributed to *NOD2/CARD15* mutations (Blau syndrome and *NOD2*-associated auto-inflammatory disease [NAID]) [6, 7]. The IVS8^+158^ variant (a C to T mutation in the intron 8 splicing region of *NOD2/CARD15*) was initially described as being more than three times as common in Ashkenazi Jewish patients with CD as in healthy controls [9], but subsequent reports of this alteration failed to confirm this association. *NOD2/CARD15* IVS8^+158^ occurs rarely in pediatric Blau syndrome, but is present in nearly all reported cases of NAID [6, 7]. The spectrum of symptoms in Case 1—including intermittent fever, polyarthralgia, and serositis (pericarditis), the presence of the *NOD2/CARD15* IVS8^+158^ mutation, and response to therapy with anti-inflammatory agents—is more consistent with an AInD than with CD. However, while pyoderma gangrenosum is a known EIM of IBD—and is characteristic of the auto-inflammatory syndrome of pyogenic arthritis, pyoderma gangrenosum, and acne (PAPA)—it has not been described in NAID. It is possible that this patient has an NAID-like auto-inflammatory disease overlapping with underlying IBD. Alternatively, the presence of the *NOD2/CARD15* IVS8^+158^ mutation may alter the phenotype of IBD or pouchitis, resulting in treatment-refractory disease with NAID-like features. In the expanded NAID case series, it was noted that patients with the R702W mutation of *NOD2/CARD15* had a high incidence of dermatitis [7], consistent with previous reports [10]. Indeed, the high frequency of R702W among the NAID cohort suggests that the combination of IVS8^+158^ and R702W might pre-dispose individuals to NAID in general [7]. The impact of other mutations of *NOD2/CARD15* (such as the 3010insC mutation in Case 1 of this report) in combination with the IVS8^+158^ variant on the clinical manifestations of AInD in ileal pouch patients, is an area for future study. For example, it would be interesting to determine whether the 3010insC mutation, in the setting of the IVS8^+158^ variant, pre-disposes individuals to chronic pouchitis or serositis as seen in Case 1. We have reported that IPAA patients with overlap of certain auto immune features (such as concurrent autoimmune thyroid disease and primary sclerosing cholangitis) are more prone to CARP [11]. A study involving larger numbers of patient is needed to determine whether the concurrence of AInD in IPAA patients similarly increases risk for CARP. In addition, it remains to be determined whether the *NOD2/CARD15* genotype influences the response to immune-modulating or anti-inflammatory therapy. AInD features in patients with underlying IBD (which is typically the case in ileal pouch patients) may require combination therapy to achieve disease control. Such an approach was used in Case 1, in which a patient with underlying CD was managed with 6-mercaptopurine as well as the cyclooxygenase 2-selective anti-inflammatory medication celecoxib.

Bowel-anatomy-altering surgical procedures, such as IPAA, may reset the body’s ‘immune thermostat', possibly via a change in the gut luminal environment. Indeed, the patient described in Case 2 presented with oral ulcers, lower extremity arthritis, and periodic papular rash in association with *NOD2/CARD15* IVS8^+158^ several years after J-pouch surgery. Other possible manifestations of altered immunity following surgery are *de novo* celiac disease [12] and *de novo* CD [13], which develop following IPAA in patients with a background of UC. Pre-disposing genetic factors, such as *NOD2/CARD15* mutations, may contribute to the initiation and/or development of the disease process after surgery. In addition, the gradual onset of AInD-related symptoms over time in the above cases may indicate the involvement of environmental factors acting as phenotypic modulators or triggers. Indeed, the observed variation in the severity of FMF following patient migration [14], as well as the phenotypic discordance rates in twins with FMF [15], indicates that the environment plays a role in AInD expression. However, the relevant environmental factors have yet to be elucidated. Tobacco smoking has a known inverse association with UC, as well as with chronic pouchitis. However, the effect of tobacco use and other environmental factors on the manifestations of AInD in ileal pouch patients is unknown and requires further study.

The AInD, including FMF, Blau syndrome, and NAID, are due to diverse genetic mutations, leading to an inflammatory cascade at multiple organ sites [3]. AInDs do not appear to be associated with auto-reactive T lymphocytes or pathogenic autoantibodies; however, there is evidence that the concepts of auto-inflammation and auto-immunity are not mutually exclusive. For example, alterations of the TNFR super family member 1A (*TNFRSF1A*) gene, the causative mutation of tumor necrosis factor receptor (TNFR)-associated periodic syndrome (TRAPS), have also been reported in systemic lupus erythematosus [16], rheumatoid arthritis [17], multiple sclerosis [18], and IBD [19]. Heterozygosity for the *MEFV* mutation responsible for FMF is associated with a higher risk for EIM and colonic strictures in CD patients (5). Our group described NAID, an AInD associated with *NOD2/CARD15* mutation, with phenotypic and genetic features overlapping Blau syndrome and CD [6, 7]. In an expanded cohort of 22 patients with NAID, most of the patients had symptoms of periodic fever, spongiotic dermatitis, polyarthritis, and serositis, and 21 (96%) had the IVS8^+128^ variant of *NOD2/CARD15* [6, 7].

The first case in this report introduces the possibility of a subclass of immune-mediated pouchitis that can be described as “pouchitis with AInD features” and suggests that these monogenetic diseases of aberrant innate immunity may play a role in the pathogenesis of chronic pouch inflammation. The symptoms of pouch inflammation in Case 1 were unresponsive to standard antibiotic regimens. The patient also had clinical features, such as fever and serositis, which are uncommon in patients with classic dysbiosis-associated pouchitis or CD. Therefore, an auto-inflammatory etiology should be suspected in IPAA patients with chronic pouchitis and a spectrum of systemic manifestations including periodic fevers, arthritis, rash, lymphadenopathy, and serositis. Genetic testing should be pursued if there is sufficient clinical suspicion for AInD. With regard to treatment, anti-inflammatory and/or immunosuppressive medications would be a consideration. In addition, colchicine is effective in the treatment of FMF [3], but has not been explored in the management of chronic pouchitis. Therapies targeting interleukin 1 show promise for the treatment of diverse AInD [3], but this option requires further study in patients with AInD and pouchitis.

## Funding

Bo Shen is supported by the Ed and Joey Story Endowed Chair. The manuscript received no direct financial support.

*Conflict of interest:* none declared.
